# Further evidence for the variability of the 18S rDNA loci in the family Tingidae (Hemiptera, Heteroptera)

**DOI:** 10.3897/CompCytogen.v10i4.9631

**Published:** 2016-10-14

**Authors:** Natalia V. Golub, Viktor B. Golub, Valentina G. Kuznetsova

**Affiliations:** 1 Zoological Institute, Russian Academy of Sciences, Universitetskaya nab. 1, St. Petersburg 199034, Russia; 2 Voronezh State University, Universitetskaya pl. 1, Voronezh, 394006, Russia

**Keywords:** Karyotype, FISH, major rDNA cluster, lace bugs, Cimicomorpha, Hemiptera

## Abstract

As of now, within the lace bug family Tingidae (Cimicomorpha), only 1.5% of the species described have been cytogenetically studied. In this paper, male karyotypes of *Stephanitis caucasica*, *Stephanitis pyri*, *Physatocheila confinis*, *Lasiacantha capucina*, *Dictyla rotundata* and *Dictyla echii* were studied using FISH mapping with an 18S rDNA marker. The results show variability: the major rDNA sites are predominantly located on a pair of autosomes but occasionally on the X and Y chromosomes. All currently available data on the distribution of the major rDNA in the Tingidae karyotypes are summarized and shortly discussed. Our main concern is to clarify whether the chromosomal position of rDNA loci can contribute to resolving the phylogenetic relationships among the Tingidae taxa.

## Introduction

The true bug family Tingidae is a relatively large and widespread group of phytophagous (sap-sucking) insects, some of which are important agricultural and forestry pests. The insects of this family are commonly known as the lace bugs due to a reticulation of the pronotum and fore wings. The family Tingidae is included in the true bug infraorder Cimicomorpha (Hemiptera, Heteroptera) and considered as the closest relative to the family Miridae, lace bugs being either placed within the superfamily Miroidea (Drake and Davis 1960, Schuh and Štys 1991, Schuh et al. 2006, 2009, etc.), or taken as an the independent superfamily Tingoidea close to the Miroidea (Scudder 1959, Štys and Kerzhner 1975, Froeschner 1996, Golub and Popov 2016, etc.)

The relationships within the Tingidae are not entirely clear (Guilbert et al. 2014). The family currently comprises approximately 2200 species classified in 280 genera (Golub and Popov 2012, Golub et al. 2012). However, chromosome sets of only 31 species (1.5%) and 17 genera (6%) are known up to now (Grozeva and Nokkala 2001, Golub et al. 2015, for other references see Ueshima 1979).

Like other Heteroptera, lace bugs possess holokinetic chromosomes characterized by a non-localised centromere (Hughes-Schrader and Schrader 1961, Ueshima 1979). In spite of several studies, the karyological evolution of the family Tingidae remains poorly understood. The lace bugs’ karyotypes seem to be highly conserved, with 12 autosomes reported for all so far studied species; the autosomes represent a series gradually decreasing in size. Most species have an XY type of sex determination while a few species have an X(0) system.

Until recently, only conventional chromosome staining techniques were used for the Tingidae. The first attempt to use a differential staining protocol was made by Grozeva and Nokkala (2001). They adapted C-banding to chromosomes of 13 species from 10 genera of lace bugs. This study revealed in karyotypes clear C-bands, which are useful for chromosome identification. Specifically, three species of the genus *Acalypta* Westwood, 1840, sharing the same karyotype of 2n = 12 + X(0), were demonstrated to differ in the number, size and location of C-heterochromatin blocks. These findings showed that C-heterochromatin distribution has had a role in the karyotype evolution of the family Tingidae.

A molecular hybridization technique such as fluorescence in situ hybridization (FISH) is a very useful method for studying molecular structure of chromosomes and differentiating separate chromosomes in different species. The chromosomal location of the rRNA genes is currently the most widely exploited marker in comparative cytogenetics of the Heteroptera (for a review see Grozeva et al. 2014). The nuclear genes coding for the ribosomal RNA are organized into the two distinct multigene families: the major rDNA repeats (genes for the 18S, 5.8S and 28S rRNAs) and the minor rDNA repeats (genes for the 5S rRNA). The major rDNA sites are often arranged in tandem arrays and undergo concerted evolution (the co-evolution of DNA sequences) being mapped to the same chromosomal region in the species karyotypes. Recently we (Golub et al. 2015) reported for the first FISH with an 18S rDNA probe in four lace bug species and discussed usefulness of the major rRNA gene cluster as a marker for revealing differences between species with similar karyotypes.

In the context of the above studies, we examined here the location of the 18S rDNA loci through FISH in six further species from the genera *Stephanitis* Stål, 1873, *Physatocheila* Fieber, 1861, *Dictyla* Stål, 1874 and *Lasiacantha* Stål, 1873. The standard karyotypes of four species, *Stephanitis caucasica*, *Stephanitis pyri*, *Physatocheila confinis* and *Dictyla rotundata* were studied for the first time.

## Materials and methods

The lace bug species used here were collected in 2015 in the Teberda Nature Reserve, North Caucasus and in Voronezh Province, Russia (Table 1). The species identification was made by V. Golub.

**Table 1.** Material used for chromosome analysis.

Species	Number of males examined	Host plant, date and locality of collection
*Dictyla echii* (Schrank, 1782)	6	*Echium* sp., 22-26.07.2015, Teberda Nature Reserve, North Caucasus, Russia.
*Dictyla rotundata* (Herrich-Schaeffer, 1835)	9	*Echium* sp., 27.07.2015, Teberda Nature Reserve, North Caucasus, Russia.
*Lasiacantha capucina* (Germar, 1837)	3	*Thymus* sp., 02.08.2015, Teberda Nature Reserve, North Caucasus, Russia.
*Physatocheila confinis* (Horváth, 1906)	3	*Crataegus* sp., 2.08.2015, Teberda Nature Reserve, North Caucasus, Russia.
*Stephanitis caucasica* Kiritshenko, 1939	12	*Rhododendron caucasicum* Pallas, 1786, 30.07.2015, Teberda Nature Reserve, North Caucasus, Russia.
*Stephanitis pyri* (Fabricius, 1775)	8	*Malus* sp., *Pyrus* sp., 15.08.2015, Voronezh Prov., Russia.

Only males were used in chromosome analysis. The specimens were fixed in the field in 3:1 Carnoy solution (96% ethanol: glacial acetic acid) and stored at 4°C. In the laboratory, testes were dissected out in a drop of 45% acetic acid and squashed on the slide. The cover slips were removed using dry ice. The preparations were stained using a Feulgen-Giemsa method by Grozeva and Nokkala (1996). To determine the number and chromosomal position of the major rDNA clusters, we carried out 18S rDNA FISH on meiotic chromosomes. In fluorescence *in situ* hybridization we followed Grozeva et al. (2014) protocol with some modifications described in Golub et al. (2015).

Chromosome slides were analyzed under a Leica DM 6000 B microscope. Images were taken with a Leica DFC 345 FX camera using Leica Application Suite 3.7 software with an Image Overlay module.

## Results

*Stephanitis caucasica*, 2n = 14 (12 + XY)

Published data: absent

**Figures 1–11.**
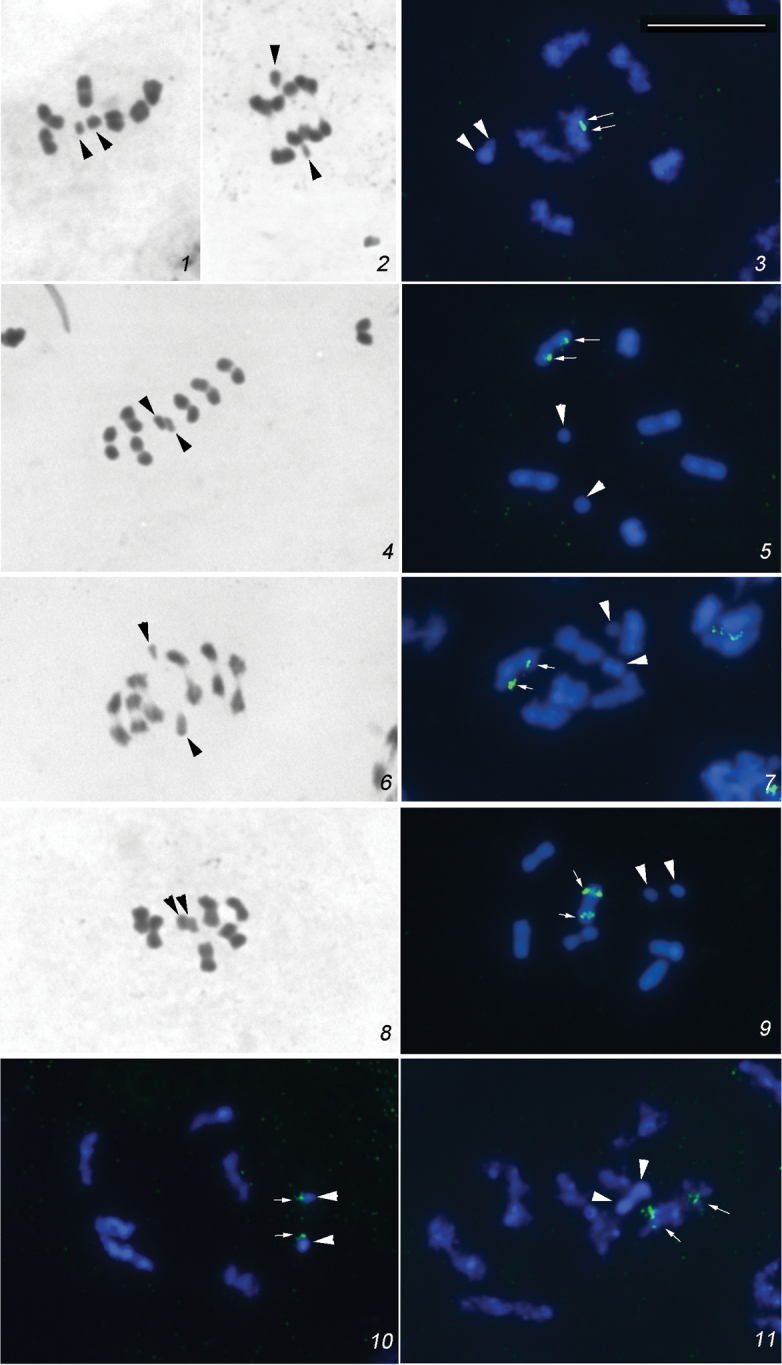
Meiotic chromosomes of the Tingidae species with 2n = 12 + XY studied using conventional staining technique and 18S rDNA FISH. **1–3** *Stephanitis caucasica* **1, 2** conventional staining: MI (1), AI (2) **3** FISH: MI **4, 5** *Stephanitis pyri* **4** conventional staining: MI **5** FISH: MI **6, 7** *Physatocheila confinis* **6** conventional staining: MI/AI transition **7** FISH: early MI **8, 9** *Dictyla rotundata* **8** conventional staining: MI **9** FISH: MI **10** *Dictyla echii* FISH: early MI **11** *Lasiacantha capucina* FISH: prophase I. rDNA FISH signals are indicated by arrows. X and Y chromosomes are indicated by arrowheads. Bar = 10µm.

At spermatocyte metaphase I (MI), six bivalents of autosomes and X and Y univalent chromosomes are present suggesting diploid karyotype of 2n = 14 (12 + XY). All bivalents are of similar size. The sex chromosomes show different sizes, the larger being presumably the X, and are situated alongside each other (Fig. 1). During an anaphase I (AI) all chromosomes undergo segregation, with X and Y chromosomes segregating ahead of the autosomes (Fig. 2).

18S rDNA FISH resulted in bright signals on an autosomal bivalent at MI. The signals are most likely located subterminally on each homolog. Sex chromosomes are placed very close to each other (Fig. 3).

*Stephanitis pyri*, 2n = 14 (12 + XY)

Published data: absent

At spermatocyte MI, six bivalents of autosomes and X and Y univalent chromosomes are present suggesting diploid karyotype of 2n = 14 (12 + XY). All bivalents are of similar size. The sex chromosomes show slightly different sizes, the larger being presumably the X, and are situated alongside each other (Fig. 4).

18S rDNA FISH resulted in bright signals on an autosomal bivalent at MI. The signals are located interstitially on each homolog. The sex chromosomes are mutually co-orientated on the spindle (Fig. 5).

*Phisatocheila confinis*, 2n = 14 (12 + XY)

Published data: absent

At spermatocyte MI/AI transition, six bivalents of autosomes and X and Y univalent chromosomes are present suggesting diploid karyotype of 2n = 14 (12 + XY). All bivalents are of similar size. The sex chromosomes show distinctly different sizes, the larger being presumably the X. The sex chromosomes segregate ahead of the autosomes (Fig. 6).

18S rDNA FISH resulted in bright signals on an autosomal bivalent at MI. The signals are located interstitially on each homolog (Fig. 7).

*Dictyla rotundata*, 2n = 14 (12 + XY)

Published data: absent

At spermatocyte MI, six bivalents of autosomes and X and Y univalent chromosomes are present suggesting diploid karyotype of 2n = 14 (12 + XY). All bivalents are of similar size. The sex chromosomes show a similar size and are situated alongside each other (Fig. 8).

18S rDNA FISH resulted in bright signals on an autosomal bivalent at MI. The signals are located interstitially on each homolog (Fig. 9).

*Dictyla echii*, 2n = 14 (12 + XY)

Published data: 2n = 14 (12 + XY) in Grozeva and Nokkala (2001)

At early spermatocyte MI, there are six bivalents of autosomes and X and Y univalent chromosomes. All bivalents are of similar size. The sex chromosomes show a similar size and are placed not far from each other. Bright 18S rDNA FISH signals are located at one end of each sex chromosome (Fig. 10).

*Lasiacantha capucina*, 2n = 14 (12 + XY)

Published data: 2n = 14 (12 + XY) in Grozeva and Nokkala (2001)

At spermatocyte prophase I, there are six bivalents of autosomes which have diffuse structure at this stage. The X and Y chromosomes are positively heteropycnotic and placed very close to each other. Bright 18S rDNA FISH signals are located interstitially on each homolog of a bivalent (Fig. 11).

## Discussion

Comparative karyotype analysis of six lace bug species was achieved using standard chromosome staining along with the 18S rDNA FISH marker. All species were found to have 2n = 14 (12 + XY). The karyotypes of *Stephanitis caucasica*, *Stephanitis pyri*, *Physatocheila confinis* and *Dictyla rotundata* were studied for the first time. The karyotypes of *Dictyla echii* and *Lasiacantha capucina* were previously studied by Grozeva and Nokkala (2001) who also reported 2n = 14 (12 + XY) for each of these species.

The results of this study confirmed the assumption of the high degree of karyotype conservation for the Tingidae (Ueshima 1979, Grozeva and Nokkala 2001, Golub et al. 2015). Including our new data, a total of 35 species from 17 genera were karyologically studied, but this represents less than 2% of known lace bug species of the world fauna. All studied species have the same number of autosomes, i.e., 12 in diploid karyotypes. The only exception might be *Acalypta parvula* (Fallén, 1807), for which different authors reported karyotypes of 2n = 12 + X(0) and 2n = 10 + XY discovered in populations from Finland and British Isles respectively (Southwood and Leston 1959, Grozeva and Nokkala 2001; for discussion, see Golub et al. 2015). Considering that Tingidae have holokinetic chromosomes, which are assumed to be susceptible to fission and fusion (Hughes-Schrader 1935), the conservation of the autosome number suggests that these rearrangements are not characteristic of lace bugs. This is supported also by the fact that in all tingid species the autosomes are of similar size, the pattern which can be considered as a ground plan feature of the family.

Despite the relative conservatism of the karyotype structure in general, some lace bug species clearly differ in size of sex chromosomes. For example, X and Y chromosomes appear noticeably heteromorphic in size in *Physatocheila confinis*, while they are evenly-sized in *Dictyla rotundata* and *Dictyla echii* (Figs 6–10). Of particular interest, detectable size differences may provide an important criterion for identification of some closely related species. For example, *Stephanitis caucasica* possesses an enlarged X chromosome in comparison to the Y, whereas in *Stephanitis pyri* both sex chromosomes appear similar in size (Figs 1–5).

Some other true bug families also demonstrate interspecies difference in size of sex chromosomes (Bardella et al. 2014, Fairbairn et al. 2016). One of the important sources of chromosome size variability seems to be related to the constitutive heterochromatin variation (White 1973). A series of lace bug species studied by C-banding was shown to differ considerably in the C-heterochromatin content and its location. Most significant variation occurs in sex chromosomes, which appear variously heterochromatin-rich in different species (Grozeva and Nokkala 2001). Although no direct information is available, the X and Y chromosome variation might be a consequence of gain and loss of heterochromatic segments during the evolution of the sex chromosomes in the Tingidae.

In the Heteroptera, the major rRNA gene FISH has yielded a significant body of literature (Grozeva et al. 2011, Panzera et al. 2012, Pita et al. 2013, Bardella et al. 2013, Chirino et al. 2013, Grozeva et al. 2014). These studies have shown that the major rDNA cluster is localized variously in tested families (reviewed in Grozeva et al. 2014). However in the Tingidae, only 10 species have been analyzed to date (Golub et al. 2015, present paper). The mapping results are summarized in Table 2.

**Table 2.** Distribution of the major rDNA loci in the Tingidae.

Species	Karyotype	18S rDNA-bearing chromosomes	The chromosomal location of 18S rDNA clusters	References
*Agramma femorale* Thomson, 1871	12 + XY	X	Subterminal	Golub et al. 2015
*Dictyla echii* (Schrank, 1782)	12 + XY	XY	Subterminal both on X and Y	Present paper
*Dictyla rotundata* (Herrich-Schaeffer, 1835)	12 + XY	AA	Interstitial	Present paper
*Elasmotropis testacea testacea* (Herrich-Schaeffer, 1830)	12 + XY	AA	Subterminal	Golub et al. 2015
*Lasiacantha capucina* (Germar, 1837)	12 + XY	AA	Interstitial	Present paper
*Physatocheila confinis* (Horvath, 1906)	12 + XY	AA	Interstitial	Present paper
*Stephanitis caucasica* Kiritshenko, 1939	12 + XY	AA	Subterminal	Present paper
*Stephanitis pyri* (Fabricius, 1775)	12 + XY	AA	Interstitial	Present paper
*Tingis crispata* (Herrich-Schaeffer, 1838)	12 + XY	X,Y*	Interstitial on X, subterminal on Y	Golub et al. 2015
*Tingis cardui* (Linnaeus, 1758)	12 + XY	AA**	Interstitial	Golub et al. 2015

*X,Y – sex chromosomes; **AA – autosomal bivalent

Despite the same chromosome number, the 18S rDNA clusters were found to vary in number (one or two in diploid karyotype) and location (sex chromosomes or autosomes) in lace bug species. The rDNA signals were observed either on the X chromosome as in *Agramma femorale*, or on both sex chromosomes as in *Tingis crispata* and *Dictyla echii*, or on a pair of autosomes as in the remaining species. The congeneric species can demonstrate both similarity and dissimilarity in the rDNA location pattern. For example, both studied *Stephanitis* species (*Stephanitis caucasica* and *Stephanitis pyri*) were found to have rDNA clusters on autosomes. A different situation arises with genera *Tingis* Fabricius, 1803 and *Dictyla*, where the congeneric species have rDNA either on autosomes or on sex chromosomes. Different mechanisms have been appointed to play a role in the rDNA evolutionary dynamics, particularly the transposition of the rRNA genes to new chromosome location in closely related species without changes in chromosome number (e.g., Granger et al. 2004, Cabrero and Camacho 2008, Nguyen et al. 2010, Panzera et al. 2012, Pita et al. 2013) and were mentioned in our previous publication (Golub et al. 2015).

Besides, the interspecific differences were found in the position of 18S rDNA clusters within chromosomes – subterminal or interstitial, and such differences are occurring likewise in congeneric species (Table 2). Specifically, subterminal clusters appeared in autosomes of *Physatocheila confinis*, *Elasmotropis testacea testacea* and *Stephanitis caucasica*; in the X chromosome of *Agramma femorale*; in the Y chromosome of *Tingis crispate*; and in both sex chromosomes of *Dictyla echii*. Furthermore, interstitial (intercalary) clusters appeared in the X chromosome of *Tingis crispata* but in autosomes of *Tingis cardui*, *Dictyla rotundata* and *Stephanitis pyri* (Table 2). Differences observed within the genus *Stephanitis* indicate that in its evolution an inversion has occurred which changed the subterminal rDNA locus in *Stephanitis caucasica* to an interstitial position in *Stephanitis pyri* or *vice versa*.

The results presented here show that the major rDNA loci in the lace bug karyotypes may be considered as essential cytological markers to compare karyotypes of phylogenetically related species and to disclose chromosomal differentiation in species with similar karyotypes. This is likewise true for the species of the subfamily Triatominae (Reduviidae) which share the karyotype of 2n = 12 + XY and show extremely high dynamics of rDNA clusters, with the variation observed both between and within the species (Panzera et al. 2012, 2014, Bardella et al. 2013, Pita et al. 2013). Because of this, the chromosomal position of rDNA loci might be a useful marker for identifying recently diverged species or populations (Pita et al. 2013).

Based on the currently available data, the autosomal major rRNA gene location appears prevalent in the Tingidae being found in 6 genera out of the 7 genera tested. The occurrence of major rDNA sites in autosomes of the Tingidae is similar to the pattern that is most frequent in the order Heteroptera (e.g., Panzera et al. 2012, Pita et al. 2013, Bardella et al. 2014, Grozeva et al. 2014). Because lace bugs have holokinetic chromosomes (without morphological markers such as centromeres), rather small chromosome size and similar karyotype structure (with all the autosomes being of similar size, so that in conventionally stained preparations the bivalents cannot be recognized on the basis of their size), it is uncertain whether an rDNA-bearing pair of autosomes is the same (homeologous) in different species. The resolution of this important issue will have to await further study based on new approaches and new discriminatory chromosomal landmarks.

In summary, the interspecific similarities and differences in the distribution of the major rDNA clusters make them promising markers for the further study of chromosome evolution in lace bugs. However, because of insufficient taxon sampling, the currently available data are inadequate to clarify the phylogenetic relationships within the Tingidae.
